# Access to pyrrolo-pyridines by gold-catalyzed hydroarylation of pyrroles tethered to terminal alkynes

**DOI:** 10.3762/bjoc.7.170

**Published:** 2011-10-26

**Authors:** Elena Borsini, Gianluigi Broggini, Andrea Fasana, Chiara Baldassarri, Angelo M Manzo, Alcide D Perboni

**Affiliations:** 1 Dipartimento di Scienze Chimiche e Ambientali Università dell’Insubria, Via Valleggio 11, 22100 Como, Italy; 2 Chemical Development, GlaxoSmithKline Medicine Research Centre, Via Fleming 4, 37135 Verona, Italy

**Keywords:** C–C coupling, gold catalysis, homogeneous catalysis, nitrogen heterocycles, rearrangement

## Abstract

In a simple procedure, the intramolecular hydroarylation of *N*-propargyl-pyrrole-2-carboxamides was accomplished with the aid of gold(III) catalysis. The reaction led to differently substituted pyrrolo[2,3-*c*]pyridine and pyrrolo[3,2-*c*]pyridine derivatives arising either from direct cyclization or from a formal rearrangement of the carboxamide group. Terminal alkynes are essential to achieve bicyclic pyrrolo-fused pyridinones by a 6-*exo*-dig process, while the presence of a phenyl group at the C–C triple bond promotes the 7-*endo*-dig cyclization giving pyrrolo-azepines.

## Introduction

Intramolecular transition-metal-catalyzed reactions represent one of the most challenging routes for the preparation of heterocyclic compounds [1–5]. Methodologies providing heterocycles, starting from readily available substrates, by different catalytic systems, as well as selective procedures for the synthesis of different heterocycles from the same starting materials by subtle modifications of the catalytic conditions, are useful tools in the hands of organic chemists. Gold catalysis has recently emerged as a suitable way to achieve such a goal, mainly thanks to the chemoselective alkynophilic properties of this attractive metal in different types of reactions [6–15]. Among the hydroarylation reactions, various kinds of heteroaryl-substituted alkynes have been demonstrated as efficient substrates for the construction of heteropolycyclic compounds. In particular, alkynyl indoles and pyrroles were successfully used to afford β-carbolines [16–17], pyrrolo-azepines [18], azepino-indoles and azocino-indoles [19] under mild conditions.

**Scheme 1:**
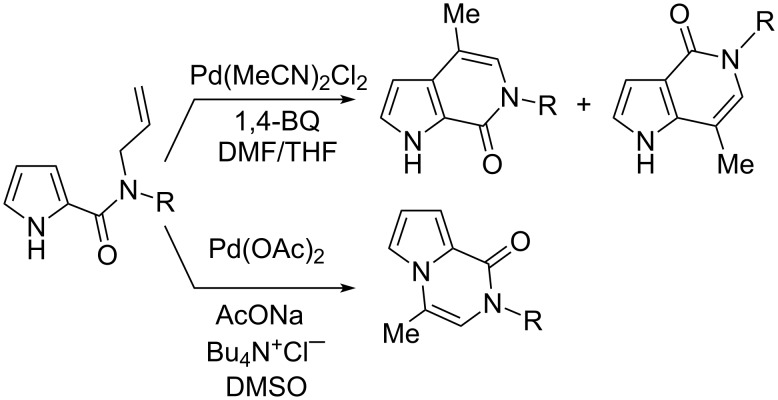
Pd-catalyzed cyclization of *N*-allyl-pyrrole-2-carboxamides.

During our studies aimed at the synthesis of complex heterocyclic systems by intramolecular transition-metal-catalyzed protocols [20–32], we reported an arylative Pd-catalyzed cyclization of *N*-allyl-pyrrole-2-carboxamides, affording pyrrolo[1,2-*a*]pyrazines or pyrrolo[2,3-*c*]pyridines (Scheme 1) [33]. The reaction provided also the isomeric pyrrolo[3,2-*c*]pyridines arising from 1,2-migration of the amide moiety. Having recently broadened our studies toward gold catalysis [34–35], we have been intrigued by the investigation of a gold-catalyzed intramolecular hydroarylation of alkynyl-tethered pyrrole-2-carboxamides in order to have an alternative protocol to access pyrrolo-fused pyridine skeletons. The importance of pyrrolo[2,3-*c*]pyridine and pyrrolo[3,2-*c*]pyridine derivatives from the biological point of view [36–50] justifies new efforts to obtain them. This study on the Au-catalyzed cyclization of *N-*alkynyl-pyrrole-2-carboxamides is also focused on the effect of the alkyne substituent, as well as on the role of the solvent on the product distribution.

## Results and Discussion

On the basis of these considerations, the *N*-propargyl amides of 1*H*-pyrrole-2-carboxylic acids **1a,b**, easily prepared in high yields from the corresponding 1*H*-pyrrole-2-carboxylic acids and *N*-methyl-*N*-propargylamine with DCC as coupling reagent, were submitted to gold-catalyzed reactions under different conditions (Table 1).

**Table 1:** Optimization of reaction conditions.

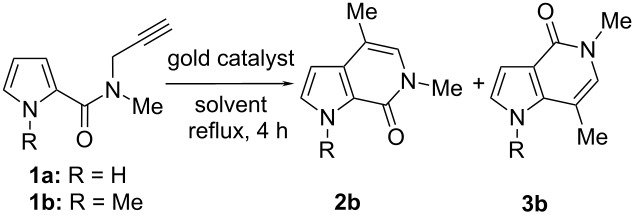					

Entry	Substrate	Gold catalyst^a^	Solvent	**2**^b^	**3**^b^

1	**1a**	AuCl_3_	MeCN	–	–
2	**1a**	NaAuCl_4_·2H_2_O	MeCN	–	–
3	**1a**	AuCl	DCM	–	–
4	**1a**	PPh_3_AuCl/AgBF_4_	DCM	–	–
5	**1a**	AuCl_3_	DCM	–	–
6	**1b**	AuCl_3_	MeCN	63	37
7^c^	**1b**	NaAuCl_4_·2H_2_O	MeCN	32	18
8	**1b**	AuCl	DCM	–	–
9	**1b**	PPh_3_AuCl/AgBF_4_	DCM	–	–

^a^Catalyst loading: 5 mol %. ^b^Determined by HPLC. ^c^In this case, 50% of **1b** was recovered.

**Figure 1:**
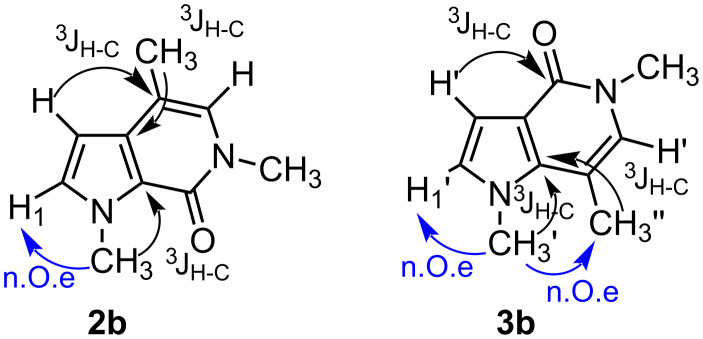
Significant relationships among hydrogen and carbon atoms arising from 2D-NMR studies to determine the pyrrolo-pyridinones.

First, it should be noted that the 1-unsubstituted pyrrole derivative **1a** did not undergo cyclization, under various conditions, based on both Au(III) and Au(I) catalysts (Table 1, entries 1–5). Otherwise, AuCl_3_ was able to promote the intramolecular reaction of the methyl-substituted substrate **1b** giving two isomeric bicyclic products. Their structures were unequivocally determined by NOESY1D, gHSQC and ^1^H/^13^C long-range correlations (gHMBC) experiments and were found to be the 6-*exo*-dig cyclization product **2b** and the rearranged structure **3b** (Figure 1). The best result was obtained when the reaction was performed in MeCN at reflux for 4 h (Table 1, entry 6); in this case, the ratio between **2b** and **3b**, determined by HPLC, was 1.7:1. The cyclization of substrate **1b** was also accomplished by working with NaAuCl_4_·2H_2_O as catalyst to give a mixture of **2b** and **3b** in the same ratio, although the conversion of the substrate was only 50% (Table 1, entry 7). Conversely, gold(I) species were unable to promote the cyclization of substrate **1b** (Table 1, entries 8 and 9).

In the presence of AuCl_3_, the ratio between the cyclization products **2b** and **3b** was significantly influenced by the solvent, as shown for the investigated conditions in Table 2. In fact, nonpolar solvents enhanced the ratio of direct to rearranged products (Table 2, entries 1 and 2), while switching to the polar ones increased the yield of **3b** up to 50% (Table 2, entry 4). In particular, toluene and DMF were proven to be the best solvents to gain the most selective conditions (7:1 versus 1:1.6, respectively).

**Table 2:** Effect of the solvent on the ratio of the isomeric products.

Entry^a^	Solvent	*T* (°C)	*t* (h)	Ratio^b^	**2b**^c^	**3b**^c^

1	Toluene	50	3	7:1	70	8
2	DCM	reflux	3	3:1	41	16
3	MeCN	reflux	4	1.7:1	45	30
4	DMF	90	4	1:1.6	32	50

^a^AuCl_3_ at 5 mol %. ^b^HPLC–MS ratio between peak areas. ^c^Isolated yields after column chromatography.

Other variously *N*-substituted propargylamides (**4a**–**d**) were submitted to the AuCl_3_-catalyzed cyclization in different solvents. Both the isolated yields and the HPLC–MS isomeric ratios of direct and rearranged products are summarized in Table 3. These reactions gave rise to a varied range of products, all having pyrrolo[2,3-*c*]pyridinone and pyrrolo[3,2-*c*]pyridinone structures. Analogously to what was observed for the substrate **1b**, benzyl- and tosyl-substituted amides **4a** and **4b** afforded the expected products **5a**,**b** and **6a**,**b**. Also in these cases, the ratio between the different products depends on the reaction solvent (Table 3, entries 1–7). In the case of the *tert*-butoxycarbonyl nitrogen-substituted substrate **4c** the reaction gave the two exo-methylene products **7c** and **8c**, with the product ratio being in the opposite sense for toluene compared to DCM (Table 3, entries 8 and 9). Unexpectedly, when working in acetonitrile, the sole product was compound **10** lacking the *tert*-butoxycarbonyl group (Table 3, entry 10). Finally, the cyclization of the benzoyl-substituted substrate **4d** furnished only products having an internal C–C double bond, with some products lacking the benzoyl group (Table 3, entries 11–14). The highest selectivity was obtained with toluene as solvent, where only pyrrolo[2,3-*c*]pyridinones, i.e., **5d** and **9** (Table 3, entry 11), were formed.

**Table 3:** Scope of the reaction on differently *N*-substituted *N*-propargyl pyrrole-carboxamides.

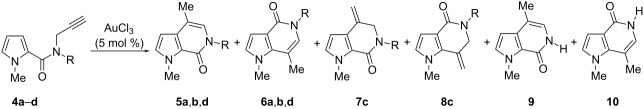												

Entry	Substrate	R	Solvent	*T* (°C)	*t* (h)	Yield (%)^a^						Ratio^b^
						**5**	**6**	**7**	**8**	**9**	**10**	

1	**4a**	Bn	Toluene	50	0.5	84	7	–	–	–	–	7.5:1
2	**4a**	Bn	DCM	reflux	0.25	61	12	–	–	–	–	3:1
3	**4a**	Bn	MeCN	reflux	0.25	50	25	–	–	–	–	1.6:1
4	**4b**	Ts	DCM	reflux	0.5	75	9	–	–	–	–	6:1
5	**4b**	Ts	Toluene	50	0.5	65	14	–	–	–	–	4:1
6	**4b**	Ts	MeCN	reflux	0.5	70	21	–	–	–	–	3:1
7	**4b**	Ts	DMF	90	7	60	25	–	–	–	–	2:1
8	**4c**	Boc	Toluene	50	1	–	–	56	14	–	–	2.5:1
9	**4c**	Boc	DCM	reflux	1	–	–	20	30	–	–	1:1.5
10	**4c**	Boc	MeCN	reflux	3	–	–	–	–	–	65^c^	1
11	**4d**	Bz	Toluene	50	1	51	–	–	–	6	–	1
12	**4d**	Bz	DCM	reflux	1.5	61	11	–	–	–	–	7:1
13	**4d**	Bz	MeCN	reflux	1	50	13	–	–	–	–	5:1
14^d^	**4d**	Bz	DMF	90	24	–	–	–	–	40	11	2:1

^a^Isolated yield. ^b^HPLC–MS ratio between peak areas. ^c^After column chromatography 33% of **4c** was recovered. ^d^At the end of the reaction 22% of **4d** was still present.

Although the picture of the products generated from the treatment of the propargyl pyrrole-2-carboxamides with AuCl_3_ as catalyst is not completely homogeneous, it should be stressed that all the substrates cyclize following a 6-*exo*-dig process. This contrasts with the outcome of the cyclization of *N*-methyl-*N*-(3-arylprop-2-ynyl)-substituted 1-methyl-pyrrole-2-carboxamides in the presence of AuCl_3_ leading to pyrrolo-azepine derivatives, as already described in the literature by Beller and coworkers [18]. In order to shed light on the structural features determining the size of the newly formed ring, we thought it appropriate to extend this study further to *N*-propargyl-1-methylpyrrole-2-carboxamides bearing a phenyl group on the acetylenic carbon. Therefore, we treated amides **11a**–**c** with AuCl_3_ as catalyst in different solvents. As given in Table 4, the reactions afforded only seven-membered cyclized products **12a**–**c** and **13a**–**c**, according to Beller’s work. This outcome confirmed the pivotal role of the substituent on the C–C triple bond to direct the cyclization following 6-*exo*-dig or 7-*endo*-dig processes. Once again, the trend in terms of direct and rearranged product formation was dependent on the solvent, the 1,2-migration products being favoured in less polar solvents. The 7-*endo*-dig cyclizations of amides **11a**–**c** were highly selective with respect to the isomerization of the newly formed carbon–carbon double bond, since it keeps its initial position in both final structures, probably due to the conjugation with the benzene ring.

**Table 4:** Cyclization reactions on differently *N*-substituted *N*-(3-phenyl-prop-2-ynyl) pyrrole-2-carboxamides.

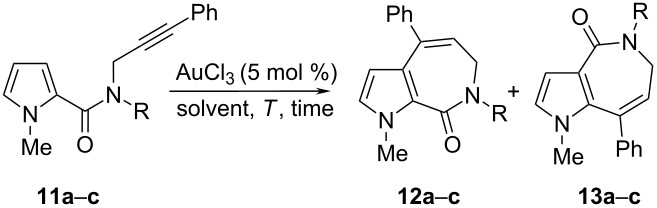								

Entry	Substrate	R	Solvent	*T* (°C)	*t* (h)	Ratio^a^	Yield^b^(%)	
						**12**:**13**	**12**	**13**

1	**11a**	Me	DCM	reflux	8	1:3	20	63
2	**11a**	Me	MeCN	reflux	2	1.5:1	43	31
3	**11a**	Me	DMF	90	24	5:1	66	12
4	**11b**	Ts	DCM	reflux	0.5	1:2.8	20	60
5	**11b**	Ts	MeCN	reflux	0.25	3:1	55	17
6	**11b**	Ts	DMF	90	24	10:1	68	7
7	**11c**	Bn	DCM	reflux	5	1:6	12	74
8	**11c**	Bn	MeCN	reflux	0.5	1:2.5	23	67
9	**11c**	Bn	DMF	90	24	5:1	66	14

^a^HPLC–MS ratio between peak areas. ^b^Isolated yields after column chromatography.

**Scheme 2:**
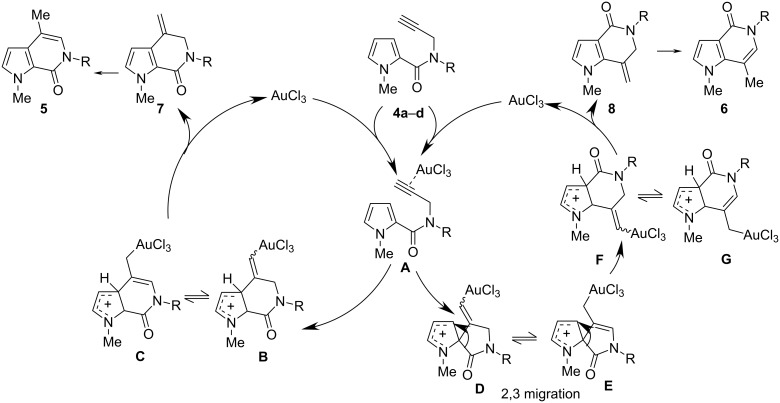
Proposed mechanism for the formation of the six-membered products.

A plausible although speculative mechanism for the hydroarylation of terminal alkynes with pyrroles is shown in Scheme 2. It is highly probable that the products arise from the vinyl gold species **B** and **F**, in turn generated from alkynes **4** after coordination to AuCl_3_. The formation of **F** can result by a migration of the acyl group from the spiro center of the transient cationic spiro gold complex **D**, in turn generated by the attack of the more nucleophilic C-2 of the pyrrole nucleus on the intermediate **A**. On the other hand, complex **B** can arise from the direct attack of the pyrrolic C-3 on the activated species **A** or from the migration of the alkyl group on the spiro intermediate **D**. The complexes **B** and **F** evolve by protodeaurylation generating the exomethylenic compounds **7** and **8**, which are eventually susceptible to alkene isomerization. The hypothesis that the alkene isomerization occurs prior to protodeaurylation, giving intermediates **C** and **E**, is unlikely because the protodemetallation is generally accepted to proceed much more rapidly on vinyl gold than on alkyl gold intermediates. In all cases, the product distribution plausibly reflects their thermodynamic stability. The endocyclic alkene is favoured presumably due to the higher degree of conjugation (compounds **5a**,**b**,**d** and **6a**,**b**,**d**), while it is reasonable to assume that the isomerization to the endocyclic alkene with the bulky *N*-BOC group (compounds **7c** and **8c**) is less favourable due to the resulting strain in the planar ring.

To explain the cyclization of alkynes **11a**–**c**, a similar mechanism could be proposed. The formation of the seven-membered cyclized products could be due to a stabilizing effect of the phenyl group in the transition state of the reaction.

## Conclusion

In summary, the cyclization of *N*-alkynyl pyrrole-2-carboxamides under very mild gold-catalyzed conditions has been described. The outcome of the intramolecular hydroarylation gave 6-*exo*-dig or 6-*endo*-dig processes, depending on the substitution of the alkyne. In both cases, products arising from a direct reaction as well as products involving a 1,2-shift of the amide group were obtained, their ratio being influenced by the polarity of the solvent. The gold-catalyzed procedure to achieve pyrrolo[2,3-*c*]pyridines and pyrrolo[3,2-*c*]pyridines represents a valuable alternative to the Pd-catalyzed cyclization of the analogue allylamides.

## Experimental

### General

Melting points were determined by capillary method with a Büchi B-540 apparatus and are uncorrected. ^1^H and ^13^C NMR spectra were recorded on an INOVA AS600 Variant spectrometer. ^13^C NMR spectra are ^1^H-decoupled and the multiplicities were determined by APT pulse sequence. Chemical shifts are given as δ values in ppm relative to residual solvent peaks (CHCl_3_) as the internal reference. IR spectra were measured with a Jasco FT/IR 5300 spectrometer. MS spectra were recorded on a HPLC–MS Agilent Technologies 6140 (ESI). Elemental analyses were executed on Perkin-Elmer CHN Analyzer Series II 2400. Preparative separations were performed by Biotage flash chromatography with 40M silica cartridges.

#### Cyclization reactions of pyrrole-carboxamides

A solution of the appropriate pyrrole-carboxamide (2 mmol) was stirred, under an argon atmosphere, with AuCl_3_ (0.01 mmol) in 30 mL of an appropriate solvent (see Table 3 and Table 4 for solvents, temperatures and times). At the end of the reaction, the solvent was either removed under reduced pressure (MeCN, DCM, toluene) or extracted with brine (DMF). The crude residue was purified by flash column chromatography.

#### 1,4,6-Trimethyl-1,6-dihydro-pyrrolo[2,3-*c*]pyridin-7-one (**2b**)

Yield: 70% (Table 2, entry 1). White solid; mp 76 °C; IR (nujol) ν: 1670 cm^−1^; ^1^H NMR (599 MHz, CDCl_3_) δ 2.13 (s, 3H), 3.49 (s, 3H), 4.13 (s, 3H), 6.17 (d, *J* = 2.9 Hz, 1H), 6.57 (s, 1H), 6.91 (d, *J* = 2.9 Hz, 1H); ^13^C NMR (150 MHz, CDCl_3_) δ 14.7 (q), 35.5 (q), 35.5 (q), 100.1 (d), 110.3 (s), 122.3 (s), 126.2 (d), 130.7 (d), 132.4 (s), 155.6 (s); MS *m*/*z*: 177.21 [M]^+^; Anal. calcd for C_10_H_12_N_2_O: C, 68.16; H, 6.86; N, 15.90; found: C, 68.22; H, 6.80; N, 15.95.

#### 1,5,7-Trimethyl-1,6-dihydro-pyrrolo[3,2-*c*]pyridin-4-one (**3b**)

Yield: 50% (Table 2, entry 4). Yellow solid; mp 104 °C; IR (nujol) ν: 1672 cm^−1^; ^1^H NMR (599 MHz, CDCl_3_) δ 2.37 (s, 3H), 3.51 (s, 3H), 3.89 (s, 3H), 6.65 (s, 1H), 6.69 (d, *J* = 3.1 Hz, 1H), 6.73 (d, *J* = 3.1 Hz, 1H); ^13^C NMR (150 MHz, CDCl_3_) δ 16.1 (q), 35.8 (q), 35.8 (q), 104.4 (d), 104.5 (s), 116.9 (s), 127.4 (d), 129.8 (d), 137.9 (s), 159.6 (s); MS *m*/*z*: 177 [M]^+^; Anal. calcd for C_10_H_12_N_2_O: C, 68.16; H, 6.86; N, 15.90; found: C, 68.13; H, 6.81; N, 15.94.

#### 1,7-Dimethyl-4-phenyl-6,7-dihydropyrrolo[2,3-*c*]azepin-8(*1H*)-one (**12a**)

Yield: 66% (Table 4, entry 3). White solid; mp 118 °C; IR (nujol) ν: 1673 cm^−1^; ^1^H NMR (599 MHz, CDCl_3_) δ 3.17 (s, 3H), 3.78 (d, *J* = 6.9 Hz, 2H), 4.00 (s, 3H), 5.95 (d, *J* = 2.8 Hz, 1H), 6.07 (t, *J* = 6.9 Hz, 1H), 6.73 (d, *J* = 2.8 Hz, 1H), 7.33–7.37 (m, 3H), 7.38–7.40 (m, 2H); ^13^C NMR (150 MHz, CDCl_3_) δ 34.6 (q), 36.6 (q), 47.5 (t), 107.7 (d), 119.6 (d), 126.6 (s), 126.7 (d), 127.1 (s), 127.7 (d), 128.1 (d), 128.5 (d), 140.5 (s), 142.6 (s), 162.2 (s); MS *m*/*z*: 253 [M]^+^; Anal. calcd for C_16_H_16_N_2_O: C, 76.16; H, 6.39; N, 11.10; found: C, 76.24; H, 6.35; N, 11.04.

#### 1,5-Dimethyl-8-phenyl-5,6-dihydropyrrolo[3,2-*c*]azepin-4(*1H*)-one (**13a**)

Yield: 63% (Table 4, entry 1). White solid; mp 130 °C; IR (nujol) ν: 1676 cm^−1^; ^1^H NMR (599 MHz, CDCl_3_) δ 3.07 (s, 3H), 3.17 (s, 3H), 3.74 (d, *J* = 7.2 Hz, 2H), 6.13 (t, *J* = 7.2 Hz, 1H), 6.63 (d, *J* = 2.8 Hz, 1H), 6.77 (d, *J* = 2.8 Hz, 1H), 7.22 (d, *J* = 6.4 Hz, 2H), 7.32–7.35 (m, 3H); ^13^C NMR (150 MHz, CDCl_3_) δ 35.3 (q), 36.4 (q), 47.5 (t), 109.6 (d), 123.2 (s), 124.3 (d), 124.7 (d), 127.5 (d), 128.0 (d), 128.6 (d), 131.5 (s), 137.8 (s), 139.3 (s), 166.0 (s); MS *m*/*z*: 253 [M]^+^; Anal. calcd for C_16_H_16_N_2_O: C, 76.16; H, 6.39; N, 11.10; found: C, 76.23; H, 6.30; N, 11.04.

## Supporting Information

File 1 Experimental procedures and characterization data.
