# Supplementary material for: Access to pyrrolo-pyridines by gold-catalyzed hydroarylation of pyrroles tethered to terminal alkynes
Source: Beilstein J Org Chem. 2011 Oct 26;7:1468–74. doi: 10.3762/bjoc.7.170 (PMC3252845; doi:10.3762/bjoc.7.170)
Supplement: File 1 — Experimental procedures and characterization data. [file Beilstein_J_Org_Chem-07-1468-s001.pdf]

Supporting Information

for

Access to pyrrolo-pyridines by gold-catalyzed hydroarylation of pyrroles tethered to terminal alkynes

Elena Borsini[1], Gianluigi Broggini*[1], Andrea Fasana[1], Chiara Baldassarri[2], Angelo M. Manzo[2] and Alcide D. Perboni[2]

Address: [1]Dipartimento di Scienze Chimiche e Ambientali Università dell'Insubria, Via Valleggio 11, 22100 Como, Italy and [2]Chemical Development, GlaxoSmithKline Medicine Research Centre, Via Fleming 4, 37135 Verona, Italy

Email: Gianluigi Broggini* - gianluigi.broggini@uninsubria.it

* Corresponding author

Experimental procedures and characterization data

Experimental

General

Melting points were determined by capillary method with a Büchi B-540 apparatus and are uncorrected. ^{1}H and ^{13}C NMR spectra were recorded on a INOVA AS600 Variant spectrometer. ^{13}C NMR spectra are ^{1}H-decoupled and the multiplicities were determined by APT pulse sequence. Chemical shifts are given as δ values in ppm relative to residual solvent peaks ($CHCl_3$) as the internal reference. IR spectra were measured with a Jasco FT/IR 5300 spectrometer. MS spectra were recorded on a HPLC–MS Agilent Technologies 6140 (ESI). Elemental analyses were executed on Perkin-Elmer CHN Analyzer Series II 2400. Preparative separations were performed by Biotage flash chromatography with 40M silica cartridges.

General procedure for the synthesis of pyrrole-2-carboxamides 1a, 1b, 4a, 11a and 11c

A mixture of the appropriate pyrrole-2-carboxylic acid (1.2 mmol) in dry CH_2Cl_2 (6 mL) was stirred at rt for 10 min under an argon atmosphere. The mixture was cooled to 0 °C and a solution of DCC (1.2 mmol) in dry CH_2Cl_2 was added dropwise while the internal temperature was kept below 5 °C. Then, the desired propargylamine (1 mmol) and DMAP (1.5 mol %) were added. The mixture was stirred at rt for 48 h, then diluted with ethyl acetate, filtered through a Celite pad, washed and rinsed with ethyl acetate. The filtrate was concentrated under reduced pressure. The crude residue was purified by flash column chromatography.

_N_-Methyl-_N_-(prop-2-ynyl)-1_H_-pyrrole-2-carboxamide (1a).

Spectroscopic data are consistent with the reported literature values [1]. Anal. Calcd. for $C_9H_{10}N_2O$: C, 66.65; H, 6.21; N, 17.27; Found. C, 66.48; H, 6.33; N, 17.05

_N_-Methyl-_N_-(prop-2-ynyl)-1-methyl-1_H_-pyrrole-2-carboxamide (1b).

Yield: 91%. Colourless oil. IR (nujol): ν = 1672 cm^{-1}. ^1H NMR (599 MHz, $CDCl_3$) δ 2.30 (t, *J* = 2.5 Hz, 1H), 3.19 (s, 3H), 3.79 (s, 3H), 4.32 (d, *J* = 2.5 Hz, 2H), 6.09. (dd, *J* = 3.9, 2.4 Hz, 1H), 6.54 (dd, *J* = 3.9, 1.1 Hz, 1H), 6.69 (dd, *J* = 2.4, 1.1 Hz, 1H). ^{13}C NMR (150 MHz, $CDCl_3$) δ 34.8 (q), 35.8 (q), 39.3 (t), 72.3 (d), 79.0 (s), 106.9 (d), 113.4 (d), 126.7 (d), 124.7 (s), 163.8 (s). MS: *m/z* 177 (M^+). Anal. Calcd. for $C_{10}H_{12}N_2O$: C, 68.16; H, 6.86; N, 15.90; Found. C, 68.32; H, 6.89; N, 15.85.

Benzyl-_N_-(prop-2-ynyl)-1-methyl-1_H_-pyrrole-2-carboxamide (4a).

Yield: 85%. Colourless oil. IR (nujol): ν = 1666 cm^{-1}. ^1H NMR (599 MHz, $CDCl_3$) δ 2.31 (br s, 1H), 3.85 (s, 3H), 4.25 (br s, 2H), 4.90 (s, 2H), 6.06–6.10 (m, 1H), 6.60 (br s, 1H), 6.71–6.75 (m, 1H), 7.28–7.32 (m, 1H), 7.31–7.38 (m, 4H). ^{13}C NMR (150 MHz, $CDCl_3$) δ 35.9 (q), 36.7 (t), 49.9 (t), 72.3 (d), 79.2 (s), 107.1 (d), 113.1 (d), 124.7 (s), 127.0 (d), 127.5 (d), 127.8 (d), 128.7 (d), 136.9 (s), 164.1 (s). MS: *m/z* 253 (M^+). Anal. Calcd. for $C_{16}H_{16}N_2O$: C, 76.16; H, 6.39; N, 11.10; Found. C, 76.21; H, 6.28; N, 11.12.

_N_-Methyl-_N_-(3-phenylprop-2-ynyl)-1-methyl-1_H_-pyrrole-2-carboxamide (11a)

Yield: 90%. Colourless oil. IR (nujol): ν = 1668 cm^{-1}. ^1H NMR (599 MHz, $CDCl_3$) δ 3.26 (s, 3H), 3.82 (s, 3H), 4.57 (s, 2H), 6.10–6.14 (m, 1H), 6.58–6.62 (m, 1H), 6.72 (s, 1H), 7.30–7.34 (m, 3H), 7.44–7.48 (m, 2H). ^{13}C NMR (150 MHz, $CDCl_3$) δ 34.9 (q), 36.0 (q), 40.5 (t), 84.5 (s), 84.6 (s), 107.2 (d), 113.6 (d), 122.9 (s), 125.1 (s), 126.8 (d), 128.5 (d), 128.6 (d),

131.9 (d), 164.0 (s). MS: *m/z* 253. (M^+). Anal. Calcd. for $C_{16}H_{16}N_2O$: C, 76.16; H, 6.39; N, 11.10. Found. C, 76.10; H, 6.37; N, 11.22.

_N_-Benzyl-_N_-(3-phenylprop-2-ynyl)-1-methyl-1_H_-pyrrole-2-carboxamide (11c)

Yield: 80%. Yellow oil. IR (nujol): ν = 1667 cm^{-1}. ^1H NMR (599 MHz, $CDCl_3$) δ 3.86 (s, 3H), 4.49 (s, 2H), 4.96 (s, 2H), 6.10 (d, *J* = 3.2 Hz, 1H), 6.67 (br s, 1H), 6.74 (d, *J* = 1.7 Hz, 1H), 7.30–7.35 (m, 4H), 7.37–7.39 (m, 2H), 7.42–7.47 (m, 2H). ^{13}C NMR (150 MHz, $CDCl_3$) δ 35.9 (q), 37.9 (t), 49.9 (t), 84.5 (s), 84.6 (s), 107.1 (d), 113.1 (d), 122.8 (s), 124.9 (s), 126.9 (d), 127.5 (d), 127.9 (d), 128.3 (d), 128.4 (d), 128.7 (d), 131.8 (d), 137.1 (s), 164.1 (s). MS: *m/z* 329 (M^+). Anal. Calcd. for $C_{22}H_{18}N_2O$: C, 80.46; H, 6.14; N, 8.53. Found C, 80.32; H, 6.21; N, 8.58.

Procedure for the synthesis of _N_-(prop-2-ynyl)-_N_-tosyl-1-methyl-1_H_-pyrrole-2-carboxamide (4b)

To a stirred solution of 1.2 g (9.59 mmol) of 1-methyl-1*H*-pyrrole-2-carboxylic acid in 21 mL of dry THF containing 2 drops of *N*,*N*-dimethylformamide, 1.25 mL (14.3 mmol) of oxalyl chloride was added. The resulting mixture was stirred at rt for 3 h; the solvent was then removed under reduced pressure and the residue taken up in 15 mL of dry THF. In a separate flask, to a suspension of 0.137 g of NaH (5.74 mmol) in 10 mL of dry THF cooled to 0 °C, 1g (4.79 mmol) of 4-methyl-*N*-(prop-2-yn-1-yl)benzenesulfonamide was added dissolved in 10 mL of dry THF by slow dropping in 15 min. The mixture was stirred at 0 °C for 30 min; then, the preformed acid chloride was added by syringe and the resulting mixture was allowed to warm to rt overnight. The mixture was quenched at 0 °C with 20 mL of water and extracted with 80 mL of ethyl acetate. The phases were separated; the organic phase was washed twice with 20 mL of water, dried over Na_2SO_4 and the solvent removed under reduced pressure. The residue was subjected to column chromatography

(cyclohexane:EtOAc 70/30) to give **4b** as a white solid (1.15 g, 77% yield). Mp 127 °C. IR (nujol): ν = 1674 cm^{-1}. ^{1}H NMR (599 MHz, $CDCl_3$) δ 2.38 (t, *J* = 2.5 Hz, 1H), 2.43 (s, 3H), 3.69 (s, 3H), 4.67 (d, *J* = 2.5 Hz, 2H), 6.13 (dd, *J* = 4.2, 2.3 Hz, 1H), 6.80 (d, *J* = 2.3 Hz, 1H), 7.06 (d, *J* = 4.2 Hz, 1H), 7.30–7.33 (m, 2H), 7.89–7.92 (m, 2H). ^{13}C NMR (150 MHz, $CDCl_3$) δ 21.6 (q), 36.2 (q), 30.1 (t), 73.1 (d), 78.9 (s), 108.2 (d), 118.9 (d), 124.1 (s), 128.7 (d), 129.3 (d), 130.7 (d), 135.8 (s), 144.6 (s), 162.1 (s). MS: *m/z* 317.37 (M^+). Anal. Calcd. for $C_{16}H_{16}N_2O_3S$: C, 60.74; H, 5.10; N, 8.85. Found. C, 60.62; H, 5.14; N, 8.88.

Procedure for the synthesis of *tert*-butyl 1-methyl-1*H*-pyrrole-2-carbonyl(prop-2-ynyl)carbamate (4c)

To a stirred solution of 0.81 g (5 mmol) of 1-methyl-*N*-(prop-2-yn-1-yl)-1*H*-pyrrole-2-carboxamide in 60 mL of acetonitrile, 1.26 g (5.8 mmol) of di-*tert*-butyl dicarbonate was added, followed by 0.061 g (0.5 mmol) of 4-dimethylaminopyridine. The resulting solution was stirred at rt overnight and the solvent removed under reduced pressure. The crude residue was subject to flash column chromatography (cyclohexane:EtOAc 80/20) to give **4c** as a colourless oil (1.1 g, 85% yield). IR (nujol): ν = 1726, 1667 cm^{-1}. ^{1}H NMR (599 MHz, $CDCl_3$) δ 1.36 (s, 9H), 2.23 (t, *J* = 2.4 Hz, 1H), 3.86 (s, 3H), 4.43 (d, *J* = 2.4 Hz, 2H), 6.10–6.12 (m, 1H), 6.60–6.64 (m, 1H), 6.80 (d, *J* = 2.2 Hz, 1H). ^{13}C NMR (150 MHz, $CDCl_3$) δ 27.7 (q), 35.0 (q), 36.0 (t), 70.9 (d), 79.3 (s), 82.8 (s), 107.9 (d), 117.8 (d), 127.5 (s), 129.4 (d), 153.1 (s), 160.2 (s). MS: *m/z* 263.30 (M^+). Anal. Calcd. for $C_{14}H_{18}N_2O_3$: C, 64.10; H, 6.92; N, 10.68. Found. C, 64.15; H, 7.04; N, 10.54.

Synthesis of 1-methyl-*N*-(phenylcarbonyl)-*N*-(prop-2-yn-1-yl)-1*H*-pyrrole-2-carboxamide (4d)

To a stirred suspension of 0.137 g (5.72 mmol) of NaH in 20 mL of dry THF a solution of 0.77 g (4.7 mmol) of 1-methyl-*N*-(phenylcarbonyl)-*N*-(prop-2-yn-1-yl)-1*H*-pyrrole-2-carboxamide (**19**) in 20 mL of dry THF was slowly added at 0 °C followed by 7 mL of dry DMF. The mixture was left under stirring at 0 °C for 0.5 h then 0.66 mL (5.7 mmol) of benzoylchloride were added. The mixture was stirred for 3 h at rt after which 10 mL of water was added followed by 30 mL of *tert*-butylmethylether. The phases were allowed to separate, the aqueous phase was discharged and the organic one was washed two times with 10 mL of water then dried over Na_2SO_4 and the solvent evaporated under reduced pressure. The crude residue was then purified by flash column chromatography (cyclohexane:cyclopentylmethylether, 6.5/3.5) to give **4d** as a colourless oil. Yield: 60%. IR (nujol): ν = 1670, 1665 cm^{-1}. ^{1}H NMR (599 MHz, $CDCl_3$) δ 2.28 (t, *J* = 2.4 Hz, 1H), 3.71 (s, 3H), 4.72 (d, *J* = 2.5 Hz, 2H), 5.92 (dd, *J* = 3.7, 2.7 Hz, 1H), 6.60 (t, *J* = 2.2 Hz, 1H), 6.62 (dd, *J* = 3.9, 1.2 Hz, 1H), 7.26 (m, 2H), 7.32 (m, 1H), 7.51 (d, *J* = 7.9 Hz, 2H). ^{13}C NMR (150 MHz, $CDCl_3$) δ 35.86 (q), 35.89 (t), 72.54 (d), 78.72 (s), 108.50 (d), 119.66 (d), 127.91 (s), 127.91 (d), 127.91 (d), 128.15 (d), 128.15 (d), 130.75 (d), 131.37 (d), 136.81 (s), 164.93 (s), 172.32 (s). MS: *m/z* 266.29 (M^+). Anal. Calcd. for $C_{16}H_{14}N_2O_2$: C, 72.16; H, 5.30; N, 10.52. Found. C, 72.04; H, 5.41; N, 10.58.

Procedure for the synthesis of *N*-(3-phenylprop-2-ynyl)-*N*-tosyl-1-methyl-1*H*-pyrrole-2-carboxamide (11b)

To a stirred solution of 0.43 g (3.5 mmol) of 1-methyl-1*H*-pyrrole-2-carboxylic acid in 8 mL of dry THF containing 2 drops of DMF, 0.45 mL (5.25 mmol) of oxalyl chloride was added. The resulting mixture was stirred at rt for 3 h; the solvent was then removed under reduced pressure and the residue was taken up in 8 mL of dry THF. In a separate flask, to a

suspension of 0.049 g of NaH (2.0 mmol) in 7 mL of dry THF cooled to 0 °C, 0.5 g (1.74 mmol) of 4-methyl-*N*-(3-phenylprop-2-yn-1-yl)benzenesulfonamide dissolved in 6 mL of dry THF was added dropwise over 15 min. The mixture was stirred for 30 min at 0 °C; the preformed acid chloride was added by syringe and the mixture was allowed to warm to rt overnight. The mixture was quenched at 0 °C with 10 mL of water and extracted with 40 mL of ethyl acetate. The phases were separated; the organic phase was washed twice with 10 mL of water, dried over Na_2SO_4 and the solvent removed under reduced pressure. The residue was subjected to column chromatography (cyclohexane:EtOAc 70/30) to give **11b** as a white solid (0.62 g, 90% yield). Mp 110 °C. IR (nujol): ν = 1668 cm^{-1}. ^1H NMR (599 MHz, $CDCl_3$) δ 2.40 (s, 3H), 3.73 (s, 3H), 4.93 (s, 2H), 6.15 (dd, *J* = 4.0, 2.4 Hz, 1H), 6.82 (d, *J* = 2.4 Hz, 1H), 7.09 (d, *J* = 4.0 Hz, 1H), 7.30–7.32 (m, 7H), 8.01 (d, *J* = 8.4 Hz, 2H). ^{13}C NMR (150 MHz, $CDCl_3$) δ 21.6 (q), 36.3 (q), 39.9 (t), 84.1 (s), 85.0 (s), 108.2 (d), 118.7 (d), 122.2 (s), 124.3 (s), 128.2 (d), 128.6 (d), 128.8 (d), 129.2 (d), 130.6 (d), 131.6 (d), 136.1 (s), 144.4 (s), 162.4 (s). MS: *m/z* 393 (M^+). Anal. Calcd. for $C_{22}H_{20}N_2O_3S$: C, 67.33; H, 5.14; N, 7.14. Found. C, 67.26; H, 5.08; N, 7.22.

Characterization of cyclized compound.

6-Benzyl-1,4-dimethyl-1,6-dihydro-pyrrolo[2,3-*c*]pyridin-7-one (5a)

Yield: 84% (Table 3, entry 1). Yellow solid. Mp 76 °C. IR (nujol): ν = 1670 cm^{-1}. ^1H NMR (599 MHz, $CDCl_3$) δ 2.18 (d, *J* = 1.3 Hz, 3H), 4.21 (s, 3H), 5.19 (s, 2H), 6.25 (d, *J* = 2.8 Hz, 1H), 6.67 (q, *J* = 1.3 Hz, 1H), 7.00 (d, *J* = 2.8 Hz, 1H), 7.26–7.30 (m, 3H), 7.32–7.34 (m, 2H). ^{13}C NMR (150 MHz, $CDCl_3$) δ 15.1 (q), 35.9 (q), 50.4 (t), 100.4 (d), 111.0 (s), 122.5 (s), 125.4 (d), 127.4 (d), 127.5 (d), 128.6 (d), 131.2 (d), 132.6 (s), 137.9 (s), 155.5 (s). MS: *m/z* 253 (M^+). Anal. Calcd. for $C_{16}H_{16}N_2O$: C, 76.16; H, 6.39; N, 11.10. Found. C, 76.21; H, 6.44; N, 11.07.

1,4-Dimethyl-6-tosyl-1,6-dihydro-pyrrolo[2,3-*c*]pyridin-7-one (5b)

Yield: 85% (Table 3, entry 4). White solid. Mp 201 °C. IR (nujol): ν = 1673 cm^{-1}. ^{1}H NMR (599 MHz, $CDCl_3$) δ 2.23 (d, *J* = 1.4 Hz, 3H), 2.43 (s, 3H), 4.01 (s, 3H), 6.21 (d, *J* = 2.6 Hz, 1H), 6.98 (d, *J* = 2.8 Hz, 1H), 7.32–7.34 (m, 2H), 7.55 (q, *J* = 1.4 Hz, 1H), 7.96–7.99 (m, 2H). ^{13}C NMR (150 MHz, $CDCl_3$) δ 15.3 (q), 21.7 (q), 36.1 (q), 101.4 (d), 111.8 (s), 119.7 (d), 121.4 (s), 128.9 (d), 129.3 (d), 132.8 (d), 133.7 (s), 135.3 (s), 144.9 (s), 153.9 (s). MS: *m/z* 317 (M^+). Anal. Calcd. for $C_{16}H_{16}N_2O_3S$: C, 60.74; H, 5.10; N, 8.85. Found. C, 60.78; H, 5.11; N, 8.77.

6-Benzoyl-1,4-dimethyl-1,6-dihydro-pyrrolo[2,3-*c*]pyridin-7-one (5d)

Yield: 61% (Table 3, entry 13). Yellow solid. Mp 93 °C. IR (nujol): ν = 1674, 1668 cm^{-1}. ^{1}H NMR (599 MHz, $CDCl_3$) δ 2.25 (s, 3H), 4.07 (s, 3H), 6.30 (d, *J* = 2.8 Hz, 1H), 7.02–7.04 (m, 1H), 7.06 (d, *J* = 2.8 Hz, 1H), 7.45–7.48 (m, 2H), 7.56–7.59 (m, 1H), 7.81 (d, *J* = 7.9 Hz, 2H). ^{13}C NMR (150 MHz, $CDCl_3$) δ 15.05 (q), 36.1 (q), 101.5 (d), 112.2 (s), 121.6 (s), 121.9 (d), 128.5 (d), 129.4 (d), 132.6 (d), 133.1 (d), 133.7 (s), 134.3 (s), 155.5 (s), 173.5 (s). MS: *m/z* 267 (M^+). Anal. Calcd. for $C_{16}H_{14}N_2O_2$: C, 72.16; H, 5.30; N, 10.52. Found. C, 72.19; H, 5.41; N, 10.46.

5-Benzyl-1,7-dimethyl-1,5-dihydro-pyrrolo[3,2-*c*]pyridin-4-one (6a)

Yield: 25% (Table 3, entry 3). White solid. Mp 106 °C. IR (nujol): ν = 1671 cm^{-1}. ^{1}H NMR (599 MHz, $CDCl_3$) δ 2.36 (s, 3H), 3.91 (s, 3H), 5.19 (s, 2H), 6.69 (br s, 1H), 6.74 (d, *J* = 3.1 Hz, 1H), 6.81 (d, *J* = 3.1 Hz, 1H), 7.26–7.30 (m, 5H). ^{13}C NMR (150 MHz, $CDCl_3$) δ 16.3 (q), 35.9 (q), 50.4 (t), 104.9 (d), 105.3 (s), 116.9 (s), 127.4 (d), 127.6 (d), 127.8 (d), 128.6 (d), 128.7 (d), 137.8 (s), 137.8 (s), 159.3 (s). MS: *m/z* 253 (M^+). Anal. Calcd for $C_{16}H_{16}N_2O$: C, 76.16; H, 6.39; N, 11.10. Found. C, 76.20; H, 6.48; N, 11.06.

1,7-Dimethyl-5-tosyl-1,5-dihydro-pyrrolo[3,2-c]pyridin-4-one (6b)

Yield: 25% (Table 3, entry 8). Yellow solid. Mp 215 °C. IR (nujol): ν = 1673 cm^{-1}. ^{1}H NMR (599 MHz, $CDCl_3$) δ 2.40 (s, 3H), 2.44 (d, *J* = 1.3 Hz, 3H), 3.90 (s, 3H), 6.64–6.68 (m, 2H), 7.31–7.33 (m, 2H), 7.59 (q, *J* = 1.3 Hz, 1H), 7.97–7.99 (m, 2H). ^{13}C NMR (150 MHz, $CDCl_3$) δ 16.7 (q), 21.6 (q), 36.2 (q), 106.0 (d), 106.6 (s), 116.7 (s), 122.7 (d), 128.1 (d), 129.3 (d), 129.3 (d), 134.9 (s), 138.1 (s), 145.2 (s), 157.4 (s). MS: *m/z* 317 (M^+). Anal. Calcd. for $C_{16}H_{16}N_2O_3S$: C, 60.74; H, 5.10; N, 8.85. Found. C, 60.61; H, 5.18; N, 8.87.

Benzoyl-1,7-dimethyl-1,5-dihydro-pyrrolo[3,2-*c*]pyridin-4-one (6d)

Yield: 13% (Table 3, entry 14). Yellow solid. Mp 165 °C. IR (nujol): ν = 1673, 1669 cm^{-1}. ^{1}H NMR (599 MHz, $CDCl_3$) δ 2.46 (s, 3H), 3.97 (s, 3H), 6.75–6.77 (m, 2H), 7.13 (s, 1H), 7.44 (dd, *J* = 7.9, 7.6 Hz, 2H), 7.56 (dd, *J* = 7.6, 7.6 Hz, 1H), 7.78 (d, *J* = 7.9 Hz, 2H). ^{13}C NMR (150 MHz, $CDCl_3$) δ 16.5 (q), 36.2 (q), 106.2 (d), 107.0 (s), 116.7 (s), 125.2 (d), 128.0 (d), 128.4 (d), 129.4 (d), 133.0 (d), 134.1 (s), 138.3 (s), 159.4 (s), 173.5 (s). MS: *m/z* 267 (M^+). Anal. Calcd. for $C_{16}H_{14}N_2O_2$: C, 72.16; H, 5.30; N, 10.52. Found. C, 72.21; H, 5.25; N, 10.49.

***tert*-Butyl 1-methyl-4-methylene-7-oxo-4,5-dihydro-1,7-dihydro -pyrrolo[2,3-*c*]pyridine-6-carboxylate (7c)**

Yield: 56% (Table 3, entry 9). White solid. Mp 92 °C. IR (nujol): ν = 1723, 1671 cm^{-1}. ^{1}H NMR (599 MHz, $CDCl_3$) δ 1.57 (s, 9H), 3.95 (s, 3H), 4.54–4.58 (m, 2H), 5.08 (t, *J* = 1.8 Hz, 1H), 5.28 (t, *J* =1.3 Hz, 1H), 6.21 (d, *J* = 2.8 Hz, 1H), 6.77 (d, *J* = 2.8 Hz, 1H). ^{13}C NMR (150 MHz, $CDCl_3$) δ 28.1 (q), 36.6 (q), 51.7 (t), 82.73 (s), 102.00 (t), 108.09 (d), 121.41 (s), 129.84 (s), 131.2 (d), 133.3 (s), 152.7 (s), 158.7 (s). MS: *m/z* 263 (M^+). Anal. Calcd. for $C_{14}H_{18}N_2O_3$: C, 64.10; H, 6.92; N, 10.68. Found. C, 64.20; H, 6.95; N, 10.61.

tert-Butyl 1-methyl-7-methylene-4-oxo-6,7-dihydro-1,4-dihydro-pyrrolo[3,2-c]pyridine-5-carboxylate (8c)

Yield: 30% (Table 3, entry 10). Colourless oil. IR (nujol): ν = 1724, 1671 cm^{-1}. ^{1}H NMR (599 MHz, $CDCl_3$) δ 1.45 (br s, 9H), 3.68 (s, 3H), 4.45 (s, 2H), 5.21 (m, 2H), 6.51 (d, *J* = 3.1 Hz, 1H), 6.55 (d, *J* = 3.1 Hz, 1H). ^{13}C NMR (150 MHz, $CDCl_3$) δ 27.87 (q), 35.8 (q), 52.4 (t), 82.3 (s), 107.6 (d), 109.6 (t), 117.4 (s), 127.0 (s), 130.2 (d), 133.5 (s), 152.7 (s), 161.7 (s). MS: *m/z* 263 (M^+). Anal. Calcd. for $C_{14}H_{18}N_2O_3$: C, 64.10; H, 6.92; N, 10.68. Found. C, 64.16; H, 6.94; N, 10.59.

1,4-Dimethyl-1,6-dihydro-pyrrolo[2,3-c]pyridin-7-one (9)

Yield: 40% (Table 3, entry 15). Yellow solid. Mp 116 °C. IR (nujol): ν = 1671 cm^{-1}. ^{1}H NMR (599 MHz, $CDCl_3$) δ 2.26 (d, *J* = 0.8 Hz, 3H), 4.20 (s, 3H), 6.34 (d, *J* = 2.8 Hz, 1H), 6.77 (br s, 1H), 7.09 (d, *J* = 2.8 Hz, 1H), 11.34 (br s, 1H). ^{13}C NMR (150 MHz, $CDCl_3$) δ 14.9 (q), 35.7 (q), 100.7 (d), 111.1 (s), 121.1 (d), 122.2 (s), 131.2 (d), 133.9 (s), 156.5 (s). MS: *m/z* 163 (M^+). Anal. Calcd. for $C_9H_{10}N_2O$: C, 66.65; H, 6.21; N, 17.27. Found. C, 66.59; H, 6.16; N, 17.32.

1,7-Dimethyl-1,5-dihydro-pyrrolo[3,2-c]pyridin-4-one (10)

Yield: 65% (Table 3, entry 11). Yellow solid. Mp 75 °C. IR (nujol): ν = 1675 cm^{-1}. ^{1}H NMR (599 MHz, $CDCl_3$) δ 2.44 (s, 3H), 3.97 (s, 3H), 6.78 (d, *J* = 3.1 Hz, 1H), 6.81 (d, *J* = 3.1 Hz, 1H), 6.82 (br s, 1H), 11.19 (br s, 1H). ^{13}C NMR (150 MHz, $CDCl_3$) δ 16.3 (q), 36.1 (q), 104.1 (d), 105.2 (s), 116.6 (s), 125.3 (d), 127.7 (d), 139.2 (s), 161.3 (s). MS: *m/z* 263 (M^+). Anal. Calcd. for $C_9H_{10}N_2O$: C, 66.65; H, 6.21; N, 17.27. Found. C, 66.63; H, 6.11; N, 17.34.

1-Methyl-4-phenyl-7-tosyl-6,7-dihydropyrrolo[2,3-*c*]azepin-8(1*H*)-one (12b)

Yield: 68% (Table 4, entry 6). White solid. Mp 190 °C. IR (nujol): ν = 1673 cm^{-1}. ^{1}H NMR (599 MHz, $CDCl_3$) δ 2.39 (s, 3H), 3.91 (s, 3H), 4.54 (br s, 2H), 5.93 (d, *J* = 2.7 Hz, 1H), 6.34 (t, *J* = 7.0 Hz, 1H), 6.78 (d, *J* = 2.7 Hz, 1H), 7.23–7.27 (m, 2H), 7.37–3.39 (m, 5H), 7.85 (d, *J* = 8.2 Hz, 2H). ^{13}C NMR (150 MHz, $CDCl_3$) δ 21.6 (q), 37.1 (q), 43.3 (t), 109.0 (d), 121.4 (d), 124.6 (s), 128.1 (d), 128.2 (d), 128.5 (d), 128.5 (d), 128.9 (s), 129.3 (d), 129.5 (d), 136.2 (s), 140.0 (s), 143.3 (s), 144.3 (s), 160.7 (s). MS: *m/z* 393 (M^+). Anal. Calcd. for $C_{22}H_{20}N_2O_3S$: C, 67.33; H, 5.14; N, 7.14. Found. C, 67.37; H, 5.19; N, 7.09.

7-Benzyl-1-methyl-4-phenyl-6,7-dihydropyrrolo[2,3-*c*]azepin-8(1*H*)-one (12c)

Yield: 66% (Table 4, entry 10). Yellow solid. Mp 106 °C. IR (nujol): ν = 1675 cm^{-1}. ^{1}H NMR (599 MHz, $CDCl_3$) δ 3.75 (d, *J* = 6.9 Hz, 2H), 4.05 (s, 3H), 4.79 (s, 2H), 5.89 (t, *J* = 6.9 Hz, 1H), 5.96 (d, *J* = 2.7 Hz, 1H), 6.76 (d, *J* = 2.7 Hz, 1H), 7.24–7.28 (m, 1H), 7.31–7.37 (m, 9H). ^{13}C NMR (150 MHz, $CDCl_3$) δ 36.80 (q), 45.2 (t), 50.3 (t), 107.8 (d), 120.2 (d), 126.8 (s), 126.8 (s), 127.0 (d), 127.2 (d), 127.7 (d), 127.8 (d), 128.0 (d), 128.5 (d), 128.5 (d), 138.0 (s), 140.5 (s), 142.4 (s), 162.3 (s). MS: *m/z* 329 (M^+). Anal. Calcd. for $C_{22}H_{20}N_2O$: C, 80.46; H, 6.14; N, 8.53. Found. C, 80.41; H, 6.18; N, 8.61.

1-Methyl-8-phenyl-5-tosyl-5,6-dihydropyrrolo[3,2-*c*]azepin-4(1*H*)-one (13b)

Yield: 60% (Table 4, entry 4). White solid. Mp 154 °C. IR (nujol): ν = 1674 cm^{-1}. ^1H NMR (599 MHz, $CDCl_3$) δ 2.40 (s, 3H), 3.06 (s, 3H), 3.88 (br s, 1H), 4.87 (br s, 1H), 6.42 (t, *J* = 7.6 Hz, 1H), 6.62 (d, *J* = 3.0 Hz, 1H), 6.71 (d, *J* = 3.0 Hz, 1H), 7.20–7.24 (m, 2H), 7.26 (d, *J* = 8.2 Hz, 2H), 7.36–7.40 (m, 3H), 7.90 (d, *J* = 8.2 Hz, 2H). ^{13}C NMR (150 MHz, $CDCl_3$) δ 21.6 (q), 36.8 (q), 43.3 (t), 110.8 (d), 121.4 (s), 125.5 (d), 126.1 (d), 127.4 (d), 128.4 (d), 128.5 (d), 128.9 (d), 129.2 (d), 132.3 (s), 136.5 (s), 138.6 (s), 138.9 (s), 144.2 (s), 164.1 (s). MS: *m/z* 393 (M^+). Anal. Calcd. for $C_{22}H_{20}N_2O_3S$: C, 67.33; H, 5.14; N, 7.14. Found. C, 67.25; H, 5.26; N, 7.06.

5-Benzyl-1-methyl-8-phenyl-5,6-dihydropyrrolo[3,2-*c*]azepin-4(1*H*)-one (13c)

Yield: 74% (Table 4, entry 8). Yellow solid. Mp 80 °C. IR (nujol): ν = 1676 cm^{-1}. ^1H NMR (599 MHz, $CDCl_3$) δ 3.08 (s, 3H), 3.70 (d, *J* = 7.3 Hz, 2H), 4.81 (br s, 2H), 5.95 (t, *J* = 7.3 Hz, 1H), 6.67 (d, *J* = 3.0 Hz, 1H), 6.85 (d, *J* = 3.0 Hz, 1H), 7.26–7.31 (m, 10H). ^{13}C NMR (150 MHz, $CDCl_3$) δ 36.5 (q), 44.9 (t), 50.6 (t), 110.0 (d), 124.8 (d), 125.1 (s), 127.1 (d), 127.5 (d), 127.9 (d), 127.9 (d), 128.0 (d), 128.4 (d), 128.7 (d), 131.5 (s), 137.8 (s), 138.3 (s), 139.5 (s), 166.0 (s). MS: *m/z* 329 (M^+). Anal. Calcd. for $C_{22}H_{20}N_2O$: C, 80.46; H, 6.14; N, 8.53. Found. C, 80.34; H, 6.25; N, 8.48.

[1] Silvestri, R.; La Regina, G.; De Martino, G.; Artico, M.; Befani, O.; Palumbo, M.; Agostinelli, E.; Turini, P. *J. Med. Chem.* **2003**, *46*, 917–920.
